# Compound impact of cognitive and physical decline: A qualitative interview study of people with Parkinson's and cognitive impairment, caregivers and professionals

**DOI:** 10.1111/hex.13950

**Published:** 2024-01-12

**Authors:** Jennifer S. Pigott, Nathan Davies, Elizabeth Chesterman, Joy Read, Danielle Nimmons, Kate Walters, Megan Armstrong, Anette Schrag

**Affiliations:** ^1^ Department of Clinical Neurosciences, Queen Square Institute of Neurology, University College London Royal Free Hospital London UK; ^2^ Research Department of Primary Care and Population Health, Centre for Ageing Population Studies University College London London UK

**Keywords:** cognitive impairment, lived experience, Parkinson's disease, Parkinson's disease dementia, qualitative

## Abstract

**Background:**

Cognitive impairment is common in Parkinson's disease and is associated with poorer quality of life and increased caregiver distress, but little qualitative information is available on lived experiences of people with Parkinson's who also have cognitive impairment.

**Objectives:**

The aim of this study was to explore the challenges of cognitive impairment in Parkinson's, triangulating the perspectives of people with Parkinson's, caregivers and healthcare professionals.

**Methods:**

Semistructured interviews were conducted with 11 people with Parkinson's and cognitive impairment, 10 family caregivers and 27 healthcare professionals, using purposive sampling in the United Kingdom (2019–2021). Cognitive impairment was identified by healthcare professionals and required subjective symptoms. Relevant cognitive diagnoses were recorded. Interviews were audio‐recorded, transcribed and analysed using reflexive thematic analysis.

**Results:**

An overarching concept of the compound impact of cognitive and physical decline was developed, with six themes. Four themes describe the experience of living with cognitive impairment in Parkinson's: (1) Challenges in Daily Activities, (2) Psychological Impact and (3) Evolving Communication Difficulties together contributing to (4) Social Shift, encompassing a reduction in wider social activities but intensification of close relationships with increased dependence. A fifth theme (5) Living Well describes positive influences on these experiences, encompassing intrinsic motivation, self‐management strategies and supportive relationships. Furthermore, underlying and shaping the whole experience was the sixth theme: (6) Preconceptions about Cognitive Impairment, describing fear and denial of symptoms and poor understanding of the nature of cognitive impairment in Parkinson's, with differences to other dementia pathologies.

**Conclusions:**

Cognitive impairment superimposed on the existing challenges of Parkinson's has a multifaceted impact and makes living with the condition arduous. Increased understanding of the experiences of this group and employing the identified facilitators for living well may be able to improve patient and caregiver experiences.

**Patient or Public Contribution:**

Two people with Parkinson's and cognitive impairment and three caregivers contributed to the study. Between them they contributed throughout the entirety of the project, giving input at conceptualisation as well as advice and review of interview questions, participant information leaflets, recruitment, interpretation of findings and summaries of the project.

## 1 INTRODUCTION

Parkinson's disease is an increasingly common^1^, ^2^ long‐term health condition with a wide range of symptoms.^3^ Cognitive impairment in Parkinson's is frequent and varied: from subjective cognitive decline and mild cognitive impairment to Parkinson's disease dementia (PDD).^4^ Longitudinal cohort studies report a 2.5–6 times higher risk of developing dementia than age‐matched people without Parkinson's of a similar age.^5^, ^6^ However, the diagnosis of cognitive impairment in Parkinson's is complex^7^ and under‐ or delayed diagnosis are prevalent.^8^ Although cognitive impairment is common throughout Parkinson's,^4^ dementia in Parkinson's is considered a hallmark of advanced disease.^9^

Parkinson's impacts quality of life and quantitative studies have shown cognitive impairment is associated with functional disability, reduced quality of life and increased caregiver strain.^10^, ^11^, ^12^, ^13^, ^14^ The clinical features and broader context in Parkinson's are heterogeneous but generally different from Alzheimer's disease, though similar to dementia with Lewy bodies (DLB).^7^, ^15^ Previous work has shown that ability to live well was even more reduced in PDD, for patients and caregivers, compared to other dementias,^16^ and caregivers of people with PDD experienced more stress than those caring for people with Alzheimer's disease and vascular dementia, and neuropsychiatric symptoms and cognitive fluctuations are associated with caregiver stress.^17^ Qualitative accounts of the lived experiences of cognitive impairment in Parkinson's are sparse. Evidence in this field is largely quantitative and focuses on family caregiver burden and unmet needs.^18^

The existing qualitative literature specific to this group has shown cognitive impairment to be perceived as a threat to identity and role in the participants with Parkinson's, with poorer adjustment.^19^ For caregivers, greater impact and feelings of loss resulted from cognitive than physical impairments in those they care for.^19^, ^20^ Lewy body dementias confer diagnostic uncertainty, fear and worry for present and future, behavioural and psychological symptoms.^18^ People with Parkinson's with executive dysfunction but *not* dementia experience difficulties in everyday life and relationships even at mild degrees of impairment.^21^

Overall, the qualitative literature on experiences of dementia is dominated by Alzheimer's disease^22^, ^23^ or dementia type is not distinguished.^24^, ^25^, ^26^, ^27^ However, experiences may be different for Parkinson's compared to other dementia processes, given the different clinical presentations and since the cognitive impairment is an evolving aspect of an existing neurodegenerative condition, with coexistent motor and nonmotor symptoms. It is therefore important to explore the perspectives of this group as distinct from other dementias.

The aim of this study was to explore the experiences of people with Parkinson's and cognitive impairment (PwP), triangulating the perspectives of people with Parkinson's, caregivers and healthcare professionals (HCPs).

## 2 MATERIALS AND METHODS

Reporting is guided by the Standards for Reporting Qualitative Research framework.^28^

### 2.1 Design

A qualitative study employing semistructured interviews and reflexive thematic analysis.^29^, ^30^

### 2.2 Patient and public involvement (PPI)

Five PPI members (two people with Parkinson's, and three caregivers) contributed to the study, all with personal experience of Parkinson's and cognitive impairment. Between them they contributed throughout the entirety of the project, giving input on conceptualisation as well as advice and review of topic guide questions, participant information leaflets, recruitment, interpretation of findings and summaries of the project.

### 2.3 Participants

Three groups of participants were recruited: PwP, family caregivers and HCPs. Clinicians in primary and secondary care identified and approached PwP in whom they recognised cognitive impairment, with or without formal cognitive testing/diagnosis. Invitation letters, information leaflets and response slips were mailed. Adverts were also circulated via relevant charities. HCP participants were identified through snowballing. Potential participants were screened for eligibility by telephone (criteria in Table 1). Purposive sampling was used to ensure diversity of different clinical and social backgrounds in terms of age, ethnicity, education, living arrangements, duration of disease, severity of functional and cognitive impairments, served by different healthcare providers (see Tables 2 and 3). For HCPs, a range of different professional backgrounds from a range of geographical areas was sought (see Table 4).

**Table 1:** Inclusion and exclusion criteria.

Sample	Criteria
People with Parkinson's and cognitive impairment	1. Diagnosis of Parkinson's disease made by a clinical specialist. 2. Cognitive impairment—Identified by the clinician and symptoms recognised by the person with Parkinson's: described in lay terms as ‘changes in memory, thinking, concentration’.^a^
	*Exclusions*: Care home residents, atypical Parkinsonian disorders and participants anticipated to be approaching end of life.
Family caregivers	A family member who closely supported the person with Parkinson's, whether or not they identified as a ‘Carer’.
	Person being supported needed to meet te inclusion criteria above.
Healthcare professionals	A person working within, or in collaboration with, healthcare, who encounters people with Parkinson's and cognitive impairment in a professional capacity.

^a^ Participants identified by a clinician as having cognitive impairment were included even in the absence of a formal diagnosis since cognitive symptoms are often underdiagnosed in clinical practice.^7^, ^8^ Participants were required to recognise cognitive symptoms since it would not be appropriate to attempt a detailed interview discussion of these symptoms if they denied them.

**Table 2:** Demographic details for people with Parkinson's and caregivers.

People with Parkinson's (interviewed directly or represented by their caregiver)	
Age	Mean 75.7 years (standard deviation 8.5 years)
	Range: 59–96 years
Sex	9 Male
	6 Female
Ethnicity	12 White British
	1 White other
	1 Asian (Indian)
	1 Black (other)
Duration of Parkinson's	Mean 13.6 years (standard deviation 6.7 years)
	Range: 2–25 years
Educational Background	Age leaving full‐time education ranged from 14 to 25 years
	Qualifications range from none, through to degrees
Schwab and England Scale^a^	Mean 47.5% (standard deviation 30%)
	Range: 10%–100%
Living arrangements	6 Live with spouse/partner
	4 With family
	5 Alone
Location	13 Urban/suburban
	1 Semirural
	1 Rural
	All from the Southeast and East of England
Caregivers	
Relationship	5 Spouse
	5 Daughter
Age	Mean 62.8 years (standard deviation 11.1 years)
	Range: 46–78 years
Sex	3 Male
	7 Female
Ethnicity	8 White British
	1 Asian (Indian)
	1 Black (other)
Location	All from the Southeast and East of England

^a^ Indicates degree of dependence, with 100% being independent and 0% being fully dependent.^31^

**Table 3:** Cognitive diagnoses of people with Parkinson's are represented.

Person with Parkinson's	Cognitive diagnosis	Nature of interview
P1	Mild cognitive impairment	Individual
P2	No formal diagnosis	Dyad + carer separately
P3	Parkinson's disease dementia	Dyad
P4	No formal diagnosis	Dyad
P5	No formal diagnosis	Individual
P6	No formal diagnosis	Dyad
P7	Parkinson's disease dementia	Carer only
P8	No formal diagnosis	Individual
P9	No formal diagnosis	Individual
P10	Parkinson's disease dementia	Carer only
P11	No formal diagnosis	Dyad
P12	Parkinson's disease dementia	Carer only
P13	No formal diagnosis	Individual
P14	Parkinson's disease dementia	Carer only
P15	Parkinson's disease dementia	Both, separately

*Note*: ‘No formal diagnosis’ indicates participants who had been identified by clinicians as having cognitive problems in the context of Parkinson's disease and subjectively reported cognitive symptoms but had not been given a formal diagnosis of dementia or mild cognitive impairment.

**Table 4:** Backgrounds of healthcare professional participants.

Professionals (*n* = 27) From the Southeast of England, the Midlands and Scotland	
6 Specialist nurses	4 Parkinson's nurse specialists
	2 Dementia nurse specialists
13 Doctors	3 Neurologists
	3 Geriatricians
	3 Psychiatrists
	3 GPs
	1 Palliative care physician
7 Allied health professionals	2 Clinical psychologists (neurological services)
	2 Occupational therapist (one Parkinson's service, one memory service)
	2 Speech and language therapists (neurological services)
	1 Physiotherapist (Parkinson's service)
1 Charity sector	1 Parkinson's UK local adviser^a^

^a^ Charity sector role to help people with Parkinson's, including providing advice and information and supporting access to services.

### 2.4 Data collection

Topic guides were developed, informed by existing literature^19^, ^21^, ^32^, ^33^, ^34^ and multidisciplinary team reviews, including PPI members, to explore experiences of living with or working with Parkinson's and cognitive impairment. Interviews were conducted by J. S. P. (clinical academic trained in qualitative research methods, not previously known to the PwP or caregiver participants and no close relationship to any HCP participants), in person, by telephone or video call, depending on public health restrictions and participant preference, November 2019–July 2021. Open questions were used to guide discussion with prompts and probes facilitated to deepen understanding. Language relating to cognitive impairment used by the participant was used by the interviewer for sensitivity. According to their preference, all PwP and caregiver interviews were conducted remotely with them in their own home, and HCPs were in their workplace. No nonparticipants were present. PwP were encouraged to ask for an interview time expected to be best for their condition, and breaks were offered to accommodate tiring.

Questions covered a range of topics (see File S1 for overview), with this article focusing on those relating to experiences of living with cognitive impairment in Parkinson's; other data has been analysed separately regarding remote consultations^35^ and healthcare services.^36^ Notes were also taken by the interviewer. Interviews were audio‐recorded and transcribed ‘verbatim’. Identifying information was removed and the accuracy of transcription was checked (J. S. P. and E. C.). Data familiarisation and preliminary analysis commenced in parallel with data collection to enable regular appraisal of ‘information power’.^37^ Data collection discontinued when the team agreed that the data had adequate power to address the study aims. A greater number of HCPs were included considering the range of different professions involved in the management of Parkinson's.

### 2.5 Analysis

Reflexive thematic analysis, described by Braun and Clark,^29^, ^30^ was used, following an inductive process. This involved data familiarisation through a thorough reading of transcripts (J. S. P. and E. C.), followed by initial creation of codes as labels of data meaning. Sample transcripts were independently reviewed, and codes were created by two other team members (M. A. and N. D.), then all codes were discussed together to agree a coding framework. Codes were iteratively developed through application to further transcripts (J. S. P. and E. C.) in NVivo 12 software^38^ with wider team review. The meaning of extracts assigned to the codes was reflected on, and themes of shared meaning created. Multidisciplinary discussion and interpretation facilitated refinement of themes. The multidisciplinary team included academics with backgrounds in geriatric medicine (J. S. P.), nursing (E. C. and J. R.), psychology (M. A., N. D. and J. R.), neurology (A. S.), general practice (D. N. and K. W.) and PPI.

## 3 RESULTS

Interviews were conducted with 11 PwP, 10 family caregivers and 27 HCPs. Although identified by clinicians and recognised by the participant, cognitive impairment frequently lacked a formal diagnosis in addition to that of Parkinson's disease (Table 3). Twenty‐seven interviews were conducted by video; 13 by telephone; four in person. Duration ranged from 41 to 121 min. Data collection predominantly took place during the coronavirus disease 2019 (Covid‐19) pandemic, with varying social restrictions in place. Experiences were drawn on from before the pandemic, but all participants discussed how it had exacerbated difficulties.

**Figure 1:**
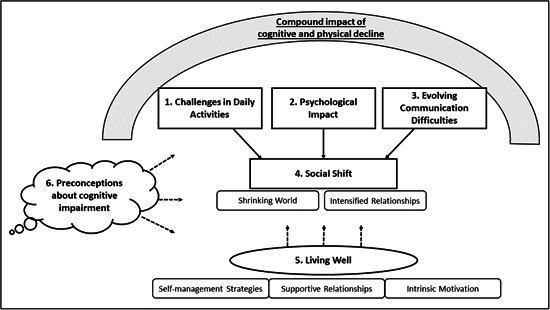
Map of themes and subthemes and their interactions. Dashed arrows indicate a modifying influence. Filled arrows indicate a causal relationship.

An overarching concept of the compound impact of cognitive and physical decline was identified. Under this umbrella, four themes were developed that described the experience of living with cognitive impairment in Parkinson's: (1) ‘Challenges in Daily Activities’, (2) ‘Psychological Impact’ and (3) ‘Evolving Communication Difficulties’ together contributing to (4) ‘Social Shift’. A fifth theme, ‘Living Well’ describes approaches to positively manage the condition, thus influencing these experiences. Furthermore, shaping the whole experience was a sixth theme: ‘Preconceptions about Cognitive Impairment’. The themes and subthemes are illustrated in Figure 1. Supportive quotes are provided in File S2.

### 3.1 Challenges in Daily Activities

All participants described the impact of symptoms on daily activities, exemplified in Table 5, explaining how the cognitive difficulties compounded the physical. Although this varied with severity of impairments, particular challenges that had evolved through the interaction of physical and cognitive decline related to mobility and falls, personal care tasks, reading and using computers.

**Table 5:** Supportive quotes for Challenges in Daily Activities.

Challenge	Supportive quote
Mobility and falls	‘… because of the Parkinson's, her memory—even on how to do things—is going away. So, for example, if she wants to stand up, she forgets that you have to do a certain number of motions to get up, or to sit down. She just gets it wrong’. (Caregiver7)
	‘Well yes, a lot of trouble—falling is a big problem … It's falling over, is the problem with my mobility … Yeah, and safety, really’. (PwP4)
Personal care tasks	‘And you are—can't do things properly. Doing things over and over again; I put on my knickers or my dress, you know, try those things, but it doesn't work properly.’ (PwP15)
Reading	‘I do find reading difficult these days. Certainly anything that really needs to be read by me seriously, I need to put it to one side so that I can remember what I need to think about or what's going on somewhere’. (PwP4)
Using computers	‘A few years ago, he was able to do email and he could look he could use the internet but now he—it's as if he's completely bamboozled by the screen. He can't, he can't work out how to, what to press’. (Caregiver2)
Remembering appointments	‘I think I've forgotten it [laugh]. Um, time. If I've got an appointment and I know in my head I've got an appointment but I have to rummage through to find out what time it was’. (PwP3)
Reduced safety awareness	‘The dementia does put her at risk, because her head is telling her to do things that she really shouldn't. […] her body still wants—well, I think it's her brain, actually, still wants to do these things, but her body is shutting down. One doesn't comprehend with the other. So, she still thinks that she can get up and walk to the front window, or go and put the kettle on, but the minute she tries to get out of that chair, she then realises, like, “oh, OK, this isn't a good idea”. And then she falls. So, it's not computing’. (Caregiver14)
Difficulty utilising resources	‘there are a lot of little gadgets around that can help you with that, like pill timers, there's all those sorts of things which are quite useful. But if your processing is not that good it might even be difficult to work out how to actually set those things’. (HCP12, PUK advisor)
Confusion over professionals involved	‘I think the other thing as well at the moment that's confusing is he's getting hospital appointments because he's got the other complaints, like he's had to have injections for his bones and hips, there's the Falls clinic, pain clinic. Then obviously he's getting new appointments and then they're cancelled, so me and my brother have taken that over now and get scolded for it because he doesn't know if he's coming or going. [to PwP:] That's really stressing you out, isn't it?’ (Caregiver6)
Managing medication	‘… with all the tablets moving about, I keep on getting confused about them’. (PwP6)
Problem solving	‘Well, I find I have difficulty writing things, working out things. I actually have to wait until then I've finished, then I go over it and see’. (PwP15)
New learning	‘I started with French when I was about 40 and started with Spanish when I was about 50. And it was enjoyable, still is enjoyable. It's a bit more frustrating now because my memory isn't as good. It's a bit more of an effort’. (PwP1)
Tiring	‘I get mentally quite tired … if I get anxious or very tired, then it all gets worse’. (PwP1)
Fluctuations	‘when she's on form she's got a good sense of humour and can chatter and all the rest of it. But when the memory's gone, which happens, then she's very quiet and very withdrawn’. (Caregiver10)
	‘When it's not working, it's—when it doesn't work, I can't—I'm like, I don't know what I'm doing. I can't—it's like, I can't do anything, not a thing at all […] I'm conscious of what I'm doing, but I just can't do them properly. So that is a big problem […] And I've got weak after that. It takes about a couple hours to wear off’. (PwP15)

Health management activities were notably impacted by the addition of cognitive impairment, even at relatively mild degrees of impairment. This manifested as difficulties in remembering appointments, reduced safety awareness, difficulty utilising resources and confusion over the multitude of different professionals involved and increasingly complicated medication regimes.

For some, cognitive impairment conveyed difficulties with problem solving or learning new things, which then impacted physical function. Many experienced physical and mental slowing and found tasks to take more effort, which caused tiredness and then further exacerbated symptoms. For some, fluctuations in medication response (physical and cognitive) impacted function.

### 3.2 Psychological Impact

Participants from all three groups consistently described negative emotional responses to the evolution of cognitive impairment on top of Parkinson's. Neuropsychiatric symptoms such as hallucinations, delusions and depression caused particular distress for PwP:

I remember people, men mainly, riding up and down my house asking me if I need to be taken somewhere. ‘Need to go for a ride?’ Now I'm not sure if these are hallucinations but that started me off with <spouse> 1 and <spouse> 2 because they were tormenting me. (PwP3)

Many participants described or perceived frustration arising from the addition of cognitive symptoms:

But my concentration is definitely gone, you know? I hate that because it's very frustrating. (PwP9)

Many HCPs and caregivers perceived PwP to struggle with emotional adjustment regarding loss of function and loss of their past roles. One described how some people lost their ‘sense of worth’ (HCP22, Parkinson's Nurse Specialist). Increasing impairments, especially cognitive, frequently conferred a feeling of losing control:

… she kept saying to us, ‘I'm going mad’. Because she could feel that her memory was declining, and she felt lack of control. (Caregiver10)

Fear and uncertainty were conveyed by many participants. Fear was expressed in relation to diagnosis and disease progression, particularly with the development of cognitive impairment:

the whole Parkinson's thing's scary [laughs]. It is. It's all such a—I never realised what a complicated condition it is. Because it really is a complicated condition. And let's face it, dementia on top of that, it's just extra difficult. Extra difficult. It's all quite scary. (Caregiver15)

Several caregivers struggled with the lack of definitive answers, unpredictability and fear of new, unexpected symptoms:

They can't say, it'll be a year, it'll be 20 years. There's no knowledge, and I think that's very scary … (Caregiver12)

### 3.3 Evolving Communication Difficulties

Communication was a profound challenge for many participants from all three groups. Physical changes in speech, gestures and facial expression were compounded by cognitive slowing:

… [speech is] very quiet and soft and they can't vocalise themselves very well. It can affect their speech in different aspects and impairment, whether it's their ability to concentrate, so in conversation their ability to search for words, you know, to express themselves, etc. So, that is difficult, as well. (HCP16, Parkinson's nurse specialist)

Loss of spontaneity of speech impacted conversation style including off‐the‐cuff comments and ability to make jokes and keeping up with conversation was problematic:

I'm not as quick in responding or in saying things. (PwP1)

For some, the combined impairments led to reliance on caregivers to support communication. This could lead to loss of privacy and autonomy for the PwP: a caregiver being required for communication impacted every interaction and anything they want to express:

[PwP] hasn't had the privacy to say anything for years. (Caregiver11)

### 3.4 Social Shift

These practical, psychological and communication difficulties led to a social shift for those with Parkinson's encompassed by two subthemes:

#### 3.4.1 Shrinking world

Participants from across the groups described PwP finding social encounters increasingly challenging due to worsening communication difficulties. Group interactions and telephone calls were especially challenging. Some PwP felt embarrassed, misunderstood or excluded:

it's embarrassing when you've been talking for five minutes and then it appears nobody heard what I've been saying. I could've been standing in the corner muttering away to myself. (PwP4)

Socialising therefore reduced, sometimes through avoidance and sometimes through social contacts not adapting to the change:

there's a tendency eventually to feeling a little bit marginal and maybe a little bit tired so you distance yourself from the group … (PwP1)

The combination of impairments was increasingly restrictive with inability to do things that were previously enjoyed. Difficulties in going out, driving, undertaking hobbies and going on holidays were common. The reduction in leisure activities and withdrawal from group socialising was conveyed as their world shrinking:

the world gets smaller, as I say, and people just do less and less and less. (HCP20, Speech and language therapist)

The lack of social activity was described by some to cause loneliness, reduce motivation and exacerbate cognitive symptoms.

#### 3.4.2 Intensified relationships

Alongside the reduction in wider social life and activities, participants described close relationships becoming more intense. The overall progression of Parkinson's caused increasing dependence, often requiring close family members to transition into and adapt to caring roles, which was challenging on both sides. For some, the onset of dementia was a pivotal moment in the perception of this role change:

I suppose it started when the dementia element came in. That's when I started to feel, yeah, I'm a carer. (Caregiver12)

The transition to caregiver and cared‐for brought negative emotions on both sides. Some PwP felt guilty or a burden, and some found themselves being short‐tempered with their caregiver:

I feel guilty, more guilty. But as I said I—I can have an out‐of‐body experience in which I watch me doing—being pernickety with what I've asked her [daughter] to do, or what she's offered to do. (PwP13)

PwP often wanted to avoid surrendering control, and displayed resistance to accepting help:

I could see he wasn't coping with the tablets, but [to PwP:] you didn't like letting go of taking your tablets, did you? (Caregiver6)

Some PwP did not feel understood by those caring for them, particularly regarding cognitive symptoms:

And my son said to me, ‘Why can't you get it into your head?’ (PwP9)

Furthermore, cognitive decline could affect insight to impairments, and differing perceptions of function between the PwP and caregiver added strain to the relationship:

… she could be confused, having a go at me, telling me she's fine when she isn't. So I do find that aspect quite difficult. (Caregiver15)

Several HCPs and caregivers highlighted specific symptoms as contributing to relationship tension: confusion, behavioural changes and apathy were especially difficult, particularly nocturnal:

I'm seeing a lot of families struggle with behaviour changes, especially if it becomes aggression, they're struggling with the aggressive behaviour. (HCP22, Parkinson's nurse specialist)

### 3.5 Living Well

Variation between individuals in terms of adjustment and coping was evident. Positive influences on living with cognitive impairment in Parkinson's were described, encompassed by three subthemes:

#### 3.5.1 Intrinsic Motivation

Different outlooks and behaviours were demonstrated. Those with Parkinson's that appeared to be coping best, typically but not universally those with milder impairments, tended to be proactive and motivated, valuing self‐reliance:

But it really comes back to me. (PwP1)

Some empowered themselves and ‘tackled’ the condition:

… he tackled the thing [Parkinson's] head on. (Caregiver11)

Some participants described a necessary adaptation process, both to Parkinson's in general and to the addition of cognitive impairment:

… the life is not over; you just have to adapt to your situation. And with my mum—so with my mum, I don't feel like she wants to adapt. She just wants to still do what she can do, which causes frustration and which causes problems, instead of maybe trying to adapt. (Caregiver15)

These individual differences in motivation and adjustment also applied to caregivers:

… I can think of two couples, and one of them, the wife just … really struggling to handle the whole diagnosis, and particularly the cognitive stuff. The other couple—very calmly she accepted it and dealt with it, and probably, his cognitive problems were worse. But I think there's—people have to get through that period of acceptance and coming to terms with what's happened. And I think that's very individual. (HCP27, physiotherapist)

Spousal caregivers were more likely to find strength in seeing the PwP and themselves as a team:

I think to myself, well, it is what it is, you're my wife, I'm here to support you and we'll go through it together. And that's what I do. (Caregiver3)

This contrasted offspring caregiver participants who appeared to struggle more with accepting deterioration of their parent.

#### 3.5.2 Self‐Management Strategies

PwP, sometimes supported by caregivers, employed a range of self‐management strategies to deal with the addition of cognitive symptoms. Several had sought information and self‐educated:

… we've done a lot of reading, [PwP] tackled Parkinson's by reading a great deal about it … he educated himself a great deal … (Caregiver11)

Being well informed appeared to facilitate better coping when new symptoms arose.

Practical strategies were employed, such as the use of daily routines, logs or diaries and technology, for example, to set reminders or alerts:

I've also got a little orange bit that comes across on my screen, it tells me when to take my tablets. That's quite good. That comes up with a little beeping noise … (PwP3)

Psychological strategies included concentrating on single tasks at one time; pacing; prioritising cognitive investment; self‐monitoring and self‐reflection:

I've just got need to be sensible myself. Not push myself too hard. Keep busy, yes, and purposeful, yes, but not try to push it too much. (PwP1)

Participants across the groups described benefits of engaging in physical and cognitive exercise, either through formal therapy or participating in hobbies and social interactions:

I'm playing bridge online, so that's one good thing that I do twice a week, so that I have to use my brain to do it. (PwP9)

#### 3.5.3 Supportive Relationships

Relationships were conveyed as key to living well with the condition. The importance of family as caregivers was impressed by nearly all participants. Detrimental effects of cognitive symptoms can be negated by support from a caregiver, and anxieties alleviated:

She [caregiver] helps with everything, because I can't do everything. I don't remember to do everything. (PwP15)

For some PwP and caregivers, peer relationships were also supportive, providing shared identity, encouragement from seeing others living well and learning from others' experiences:

I think it's important for folks to get together … And you say to, ‘I've tried this and this is working’ … and to know what people actually feel. (PwP9)

However, peer support did not suit everyone: others had negative experiences, finding it ‘*depressing*’ to see what could happen or feeling ‘*guilty*’, perceiving others’ conditions to be worse.

Maintenance of social networks conferred better quality of life for many:

she [PwP] has company as much as possible. Then she's in a better frame of mind and, therefore, can do things better. (Caregiver7)

### 3.6 Preconceptions of Cognitive Impairment

‘Dementia’ had widespread negative connotations across the participant groups and the word conferred fear in most PwP and caregivers. The distinction between Parkinson‐related cognitive impairment and other cognitive conditions was perceived by many to be poorly understood, including by professionals.

Many HCPs perceived stigma around dementia to engender denial and make conversations about cognition challenging:

… you have to be very careful how you word it around the stigma of dementia … (HCP22, Parkinson's nurse specialist)

Frequently, cognitive decline was not acknowledged, and many described a state of denial, predominantly driven by fear. There was evidence of PwP and some caregivers distancing the term ‘dementia’ and minimising cognitive symptoms within the interviews, and several HCPs described this from their experience:

…. I don't think he's got any dementia or anything like that. Just, on occasions, he forgets. (Caregiver4—followed by an extensive description of cognitive impairment impacting daily life)

Some PwP were more aware of their denial, describing how they ignored cognitive symptoms, some referencing personal observations of ‘dementia’ in explaining their concerns:

I've intentionally tried to keep away from that because my mother died of vascular dementia … I just don't want to think in that direction at all so I tend to ignore it, I know it won't go away but … (PwP5)

For some, fear was a barrier to assessment and therefore diagnosis:

No. I turned it [cognitive assessment] down. […] I think, at the time, I was afraid I was getting worse, and I feared what was going to happen if they found—which I suspect they would find—early stages of dementia. (PwP13)

Some participants commented that cognition was not always addressed by HCPs:

I don't often hear people [professionals] offer to say, ‘do you have any problems with cognition or memory and would you like to do X or Y?’. I haven't often heard that. (PwP1)

This was sometimes attributed to lack of time or capacity, or lack of HCPs' knowledge. Many HCPs described cognitive impairment in the context of Parkinson's to be a further loss, and a dementia diagnosis to be ‘devastating’ (HCP3, nurse practitioner, memory service), which could be a barrier to discussion:

The fact that people might develop dementia might be the final straw that people don't want to talk about … I think with Parkinson's in particular, it's a chronic disease and the dementia it's, you know, it's another kind of added on terrible thing to happen really. (HCP2, psychiatrist)

In some cases, cognitive changes go unnoticed or are noticed but misattributed. Many HCPs reported that cognitive decline was often blamed on ageing, and indeed age was referenced by several PwP and caregiver participants:

… but all 60‐year‐old's memories deteriorate a bit. (PwP8)

Some participants perceived a poor understanding of the nature of cognitive changes in Parkinson's to contribute to this:

… people think of dementia as being a memory problem. Whereas in dementia in Parkinson's it may be more executive functioning, so may be more around organisation or planning or visuo‐spatial problems so it may not always be obvious … (HCP2, psychiatrist)

## 4 DISCUSSION

Our study highlights the importance of *compound* cognitive and physical deficits that underpin the lived experience, through a variety of facets as illustrated in Figure 1. There has been a paucity of qualitative research in this specific patient group, yet the distinction from other cognitive pathologies and added challenge of cognitive problems within Parkinson's were perceived as important by many of our study participants.

### 4.1 Context of previous research

Findings were consistent with the small body of literature in this patient group as discussed already. Comparing our findings to those of overlapping but different patient groups highlights subtle differences in experiences. Everyday task difficulties, emotional struggles, communication problems and social changes, are all reported both in dementia^22^, ^25^, ^39^, ^40^ and in Parkinson's (particularly late stage)^19^, ^41^, ^42^, ^43^, ^44^ more broadly. The important distinction in the present study was the emphasis on the *compound* effect of cognitive and physical symptoms on each of these aspects. Slowing, tiredness and fluctuations can also be both physical and cognitive, complicating living with and managing impairments. Motor and nonmotor fluctuations are well recognised in Parkinson's,^45^, ^46^, ^47^ with profound impact of motor fluctuations reported,^43^ but the lived experience of cognitive fluctuations in Parkinson's has not been previously reported to our knowledge. The notable distress caused by neuropsychiatric symptoms is important given the marked prevalence of these symptoms in this patient group, as seen in quantitative studies.^19^, ^48^, ^49^, ^50^ People with Parkinson's *and* and cognitive impairment lose their voice both physically and metaphorically, from cognitive change and the associated shift toward communication via caregivers, with marked impact on relationships.

The difficulties identified with health management even at relatively mild degrees of cognitive impairment are important to recognise in this patient group, given the clinical complexity of Parkinson's. For example, complex dynamic medication regimes for motor fluctuations will be harder to implement when memory, learning and processing are impaired, heightened by communication difficulties between PwP and clinicians. This requires careful clinical consideration and potentially additional support.

Experiences of dementia and Parkinson's more broadly have been conceptualised as ‘loss’.^19^, ^22^, ^41^ In the present study, cognitive impairment was perceived as a major additional loss, for some the ‘final straw’, developing on the background of established physical, emotional and social losses. This marks Parkinson‐related cognitive impairment out from other dementia pathologies: a new dimension to existing decline, rather than a new condition. Moreover, fear of acknowledging that further loss was a potential barrier to discussing cognitive concerns.

We found uncertainty to be a source of fear for many PwP and caregivers. This related to how symptoms would exhibit day to day, and to the evolution of symptoms over time with fear around prognosis, both of which have been highlighted in Parkinson's more broadly.^41^, ^51^, ^52^ Fear and uncertainty around *dementia* were however marked for this patient group. Dementia as a whole is shrouded in uncertainty^53^ and unpredictability of the disease course.^54^, ^55^ This again highlights the composite effects of the Parkinson's with cognitive decline. More positively, whilst some uncertainty was considered unavoidable, the distress from unexpected symptoms could potentially be ameliorated with information provision.

Self‐management strategies, akin to previously reported ‘problem focussed’ coping strategies,^19^ were employed across the cognitive spectrum. They included practical and psychological strategies as well as information seeking and exercise or stimulation. Cognitive impairment has often been used as exclusion criteria in studies of self‐management in Parkinson's,^56^ but the experiences of our participants suggest this warrants further investigation. As for dementia^25^, ^57^, ^58^, ^59^ and Parkinson's more broadly,^60^ relationships within families and wider social networks were also facilitators to better wellbeing. However, the dual impairments for this patient group may heighten barriers to peer support compared to other dementias or Parkinson's without cognitive impairment. For example, visible physical changes from Parkinson's impact self‐perception, and slowed cognitive processing impacts social interaction.

Consistent with global reports^61^ and a study of both Alzheimer's disease and Parkinson's,^62^ negative perceptions and stigma, particularly around dementia, were perceived to be widespread. Whilst not widely explored in Parkinson's literature, diagnostic difficulty resulting from deficits being hidden has been reported by HCPs in dementia care,^63^ and is important for clinicians to be aware of. As for DLB,^64^ poor understanding of this subtype of cognitive impairment was a perceived issue, in part due to the prominence of nonamnestic cognitive changes. Poor awareness of the heterogeneity of dementia by wider society^64^ was echoed in our study.

### 4.2 Implications

This study has shown some broad similarities between experiences of cognitive impairment in Parkinson's and other cognitive pathologies but also highlighted differences considered important for clinical practice and public awareness. Table 6 summarises these findings with applications and implications.

**Table 6:** Summary of findings and implications.

Theme and subthemes	Summary of findings	Implications and application
Challenges in Daily Activities	Across the cognitive spectrum, cognitive impairment compounds physical impairment conferring difficulties in daily activities, including health‐management tasks Tiring exacerbates this Fluctuations challenge function	Those involved should seek to support strategies to deal with both aspects of the illness and the impact on activities of daily living, including fluctuations—within healthcare this may involve a multidisciplinary review Additional support may be needed to manage health and engage with healthcare and complex management requires careful consideration of cognition and recognition of fluctuations
Psychological Impact	Distressing symptoms may evolve, particularly neuropsychiatric Frustration, feelings of loss and loss of control can result Uncertainty with unpredictable symptoms and progression, particularly around ‘dementia’, generates fear	Neuropsychiatric symptoms should be explored and where possible treated Psychological support should be provided Information provision to PwP and caregivers may ameliorate some difficulties with uncertainty Addressing cognitive change, clinicians should be aware of the perception of it as an additional ‘loss’ and the potential for this to be a barrier to discussion
Evolving Communication Difficulties	Physical and cognitive symptoms both contribute to communication difficulties PwP finds it hard to keep up with conversation and maintain spontaneity of speech Increased reliance on caregivers and loss of the PwP's voice	Communication strategies and support for PwP are important Those involved in care should also be taught communication approaches Clinicians should endeavour to hear both PwP and caregiver voices
Social Shift	Resultant reduction in social life, particularly group contexts Increased dependence on people close to them Transition into cared‐for and caregiver is challenging on both sides	Communication strategies and psychological support as above and wider understanding as below, may be able to ameliorate some of the social withdrawal seen Caregivers should be supported with the increasing intensity of their role and provided information on strategies for dealing with difficult symptoms
Shrinking world		
Intensified relationships		
Living Well	Being proactive and motivated, valuing self‐reliance and adapting to circumstances seemed to be associated with better coping Information, practical and psychological strategies and stimulating and social activities appear beneficial Supportive relationships were important for overall wellbeing	Intrinsic motivation (of PwP and caregivers) should be embraced Self‐management for PwP and those supporting them may be helpful so should be supported, though further research is warranted Stimulating and social activities should be encouraged, with consideration to other aspects of their condition Supportive relationships should be fostered—and caregivers supported to continue their role Peer support should be signposted, but personal preferences recognised
Intrinsic Motivation		
Self‐Management Strategies		
Supportive Relationships		
Preconceptions about Cognitive Impairment	Dementia is stigmatised Fear and denial arose regarding cognitive decline Nonmemory symptoms associated with cognitive decline in Parkinson's may be less well understood or recognised	Greater awareness of cognitive impairment in Parkinson's disease is needed amongst clinicians, caregivers and the public, including:
		▪ Stigma can be a barrier to assessment and acceptance ▪ Understanding the context of existing diagnosis and impairments ▪ Differences to other dementia processes—different cognitive profile, trajectory and associated symptoms

### 4.3 Strengths and limitations

A range of individual demographic and disease factors and professional backgrounds were represented in this research. Conducting the study remotely enabled inclusion from a wider geographical area. However, the patient and caregiver participants were all based in the Southeast and East of England, more were from urban and semiurban than rural areas, and there is a lack of non‐English language‐speaking participants. Experiences may differ in these contexts, so some perspectives are potentially underrepresented. This concern is somewhat ameliorated by the inclusion of HCPs with a breadth of experience from different settings and patient groups. The consistency between HCP and PwP/caregiver experiences suggests that the presented findings are relatively transferable within the United Kingdom, though more subtle differences relating to these factors were not explored. Inclusion of participants with subjective cognitive symptoms rather than a formal diagnostic process prevented being restricted by the problem of underdiagnosis.^8^ Conversely, we did not conduct cognitive testing or formal assessment of Parkinson's severity, so findings cannot be interpreted in the context of these objective measures. The multidisciplinary research team and PPI input throughout helped with interpretation of data and meaning for practice and policy. An unavoidable challenge for research with this population, as with dementia,^65^ is that the condition conveys communication difficulties. Some participants had difficulty expressing their views and caregivers proxy views could be biased. The use of remote interviews may have exacerbated communication difficulties for some but were necessary due to public health restrictions in the context of Covid‐19 or were participant preferences. Individuals with more severe impairments but no caregiver may be underrepresented.

## 5 CONCLUSIONS

Cognitive impairment superimposed on the existing symptoms of Parkinson's has a multifaceted impact on life: daily activities, psychological wellbeing and communication are all affected. Together these lead to a ‘shrinking’ of the world in which the person lives and intensified close relationships. Intrinsic motivation, self‐management strategies and supportive relationships have the potential to modify the experience for the better. Poor understanding of cognitive impairment in Parkinson's, compounded by fear and stigma around dementia leads to underrecognition. We identified a need for a more nuanced understanding of this complex condition, in which the physical and cognitive aspects combine and shape the experience of people living with Parkinson's and cognitive impairment. The findings should be used to deepen understanding in those who support PwP and cognitive impairment and promote wider awareness, tackling stigma and fear.

## AUTHOR CONTRIBUTIONS

**Jennifer S. Pigott:** Conceptualization; methodology; investigation; formal analysis; funding acquisition; project administration; writing—original draft; data curation. **Nathan Davies**: Conceptualisation; methodology; supervision; writing—review and editing; formal analysis; funding acquisition. **Elizabeth Chesterman**: Formal analysis; validation; writing—review and editing; data curation. **Joy Read**: Methodology; formal analysis; writing—review and editing; project administration. **Danielle Nimmons**: Methodology; formal analysis; writing—review and editing; funding acquisition. **Kate Walters**: Conceptualisation; methodology; formal analysis; writing—review and editing; supervision; funding acquisition. **Megan Armstrong**: Formal analysis; writing—review and editing; supervision; funding acquisition. **Anette Schrag**: Conceptualisation; methodology; formal analysis; supervision; funding acquisition; writing—review and editing.

## CONFLICT OF INTEREST STATEMENT

The authors declare no conflict of interest.

## ETHICS STATEMENT

This study was approved by the London Queen Square Research Ethics Committee (18/LO/1470) and Health Research Authority approval. All participants provided written or audio‐recorded verbal informed consent.

## Supporting information

Supporting information.

Supporting information.

## Data Availability

All supporting data is included in the article. The full transcripts are not available publicly due to ethical requirements.
