# Supplementary material for: Compound impact of cognitive and physical decline: A qualitative interview study of people with Parkinson's and cognitive impairment, caregivers and professionals
Source: Health Expect. 2024 Jan 12;27(1):e13950. doi: 10.1111/hex.13950 (PMC10785559; doi:10.1111/hex.13950)
Supplement: Supplementary file 1 — Supporting information. [file HEX-27-e13950-s002.docx]

**Interview Guide for People with Parkinson’s**

1. **Current condition and management**

*Parkinson’s in general, and cognitive impairment:*

- Overall perception
- Difficulties
- Current management
- Facilitators
- Barriers
- Others consulted

1. **Current help**

- Sources and experience of support
- *Parkinson’s in general, and cognitive impairment*
- Most & least helpful
- Unmet need
- What optimal support would be

1. **Experience and views of remote support**

- Telephone
- Internet/online
- Healthcare

**Interview Guide for Caregivers**

1. **Current situation**

- Role
- Difficulties/challenges
- *Parkinson’s in general, and cognitive impairment*
- Barriers to optimal management

1. **Maintaining wellbeing and independence**

- Facilitators for person with Parkinson’s
- Facilitators for caregiver

1. **View on help, information and resources**

- What is needed?
- *Parkinson’s in general, and cognitive impairment*
- Experience of existing information/resources

1. **Experience and views of remote support**

- Telephone
- Internet/online
- Healthcare

**Interview Guide for Healthcare Professionals**

1. **Role & Experience**
2. **Difficulties in management**

(Parkinson’s + cognitive impairment)

1. **Reports from patients and caregivers**
2. **Signposting**
3. **How support could be best provided**
4. **Remote consultations**
