# Supplementary material for: Compound impact of cognitive and physical decline: A qualitative interview study of people with Parkinson's and cognitive impairment, caregivers and professionals
Source: Health Expect. 2024 Jan 12;27(1):e13950. doi: 10.1111/hex.13950 (PMC10785559; doi:10.1111/hex.13950)
Supplement: Supplementary file 2 — Supporting information. [file HEX-27-e13950-s001.docx]

1. **Challenges in Daily Activities**

| **Challenge** | **Additional Supportive Quote** |
| --- | --- |
| Mobility and falls | *“…or people see things in a very different way, and that that then is also meaning that perhaps navigating an environment in terms of their mobility is also more difficult if their visuospatial perception is impacted. So, I think it’s, kind of, that knock on effect of cognitive emotional and how that impacts on somebody’s day to day activity and the resources and the energy resources they’re able to then do.” HPC23 (psychologist)* |
| Personal care tasks | *“It’s quite difficult with Parkinson’s Disease; it’s very difficult to do it… And you are – can’t do things properly. Doing things over and over again; I put on my knickers or my dress, you know, try those things, but it doesn’t work properly” PwP15* |
| Reading | *“Because invariably people will say, “I read a sentence and then I have to go back and read it again because I can't remember what I was reading.” Or, “I get distracted, or I lack motivation in reading something that’s not really interesting to me.”” HCP12 (PUK Adviser)* |
| Using computers | *“I was doing some stuff this morning, getting a bit frustrated with my computer, was going off doing this and doing that, and I’m just not as competent at doing things, even clicking and remembering and putting things together.” PwP1* |
| Remembering appointments | *“But I mean today I went for a home visit and the lady has been reminded and sent a letter, well luckily I caught her, but she was just going out of the door… So you know even if you go to someone’s home they don’t remember you’re coming.” HCP2 (Psychiatrist)* |
| Reduced safety awareness | *“I’m just thinking again of a similar person who was saying, “I’ll be fine to go home because I’ll cope when I get there. I don’t need that much help from my wife, I can make my own meals,” and that sort of thing, when actually they just were not able to. So I think the cognitive aspects of knowing their safety, safety awareness as well and being able to manage at home.” HCP10 (Geriatrician)* |
| Difficulty utilising resources | Regarding Parkinson’s charity information: *“I do feel in a way that maybe I don’t read everything that comes through the letterbox, because – not because I don’t want to read it or because it’s not getting through to me, but it’s just sometimes … I think that I’m sort of loading… sort of messing around and doing things a lot to [spouse] and opting out of things that I couldn’t do myself.” PwP4* |
| Confusion over professionals involved | *“But that is definitely my experience that people find it quite confusing, they have too many people involved. And you may have seen a neurologist the week before and then see someone in the memory service, and you may have had the same review but you don’t realise. Asking the same questions.” HCP2 (psychiatrist)* |
| Managing medication | *“Well … in taking them, really – in taking the tablets and all that. But we have a- it’s taking them, I might not be taking the right one, or at the right time.” PWP15* |
| Problem solving | *“He [PwP] just doesn’t have the resources to look up the phone number or to use the phone. He can use the phone, he will phone people that he knows but it would be very difficult for him to handle his own affairs.” Caregiver2* |
| New learning | *“…they struggle with, problem solving, planning, that sort of thing… I think because of the executive function, learning new things is quite difficult for them.” HCP25 (OT, Parkinson’s service)* |
| Tiring | “*my brain would be exhausted” PwP8* |
| Fluctuations | *“…when it [medication] doesn’t work, I can’t – I’m like, I don’t know what I’m doing. I can’t – it’s like, I can’t do anything, not a thing at all.” PwP15* |

1. **Psychological Impact**

*“depression is quite a horrible thing” PwP9*

*“to say I’m managing it would be a bit of an overstatement. I mean I’m being managed by it [condition]” PwP2*

*“I think the two patients I have in mind, they were previously very high functioning, and I think they were aware that their memory is slipping away and that creates a lot of frustration. […] he was having very vivid visual hallucination, and that was distressing for him as well as for the wife, and we just couldn't resolve that. Like whatever medication we tried it just wasn’t treating it, and that was very distressing… and when he was having these visual hallucination he was so distressed he would scream” HCP15 (GP)*

*“And, it’s also that message of, this may be something that’s affected for you, but we don’t know if it will be, and we don’t know how much. Again, the uncertainty comes in there and it’s naturally a difficult thing for people to process” HCP9 (Psychologist)*

*“it’s upsetting them as well to understand and realise what they've become. Because I think that’s the thing as well, isn't it, it’s the realisation that they can’t do anything for themselves, or hardly anything, that’s how they think.” HCP26 (PUK Advisor)*

*“he was such a practical person. And now he can’t really do any of it. It’s dangerous to let him try […] But it [investment] was something he was very, very good at, which is why we’re comfortably off. So, everything he was good at has been taken away from him by the disease.” Caregiver12*

*“It’s only through like living with it now that I understand it a bit better, but I wouldn't have put two and two together, that was never going to happen, the freezing through doorways and knowing paths and stuff. It’s quite scary the first time really, isn't it?” Caregiver6*

*Asked what aspects were difficult: “Not knowing how my mum is going to be.” Caregiver15*

1. **Evolving Communication Difficulties**

*“people might struggle with longer complex sentences from a comprehension point of view… And then, there’s the switching, isn’t there, that’s the other challenge that in conversation when they’re talking somebody might be talking about one topic, they’ll still think about that topic and then they move to another topic and that individual is still thinking about the topic that they were just talking about. […] because of the multitasking dropping off in Parkinson’s, people tend to stop gesturing. And, gesture, as well, being really helpful for turn taking, you know, people can then see that you’re about to come into conversation and that kind of thing. […] Quite often people are taking a back seat in conversation, that they’re not talking as much, that they feel talked over, that people aren't listening to them, they feel left out and it then makes them feel like they’ve got more of a disability.” HCP20 (SLT)*

*“I suppose sometimes they’re a bit harder to read, people who have like an expressionless face. But yeah, it’s just about trying to engage as much as possible. Sometimes you might think they’re a bit rude, but you learn from experience, don’t you?” HCP7 (Nurse, Memory Service)*

*“You know, actually, that’s sometimes quite a big impact that spontaneity feels like it’s gone, timing feels like it’s gone. Some people might be mostly related to people through jokes, you know…[…] There’s no off the cuff comments, because you can’t understand them so, you know, that can feel quite hard going” HCP20 (SLT)*

*“So yes, it’s the thinking that slows down, isn't it? Added to the fact that it takes such an effort to say it.” Caregiver11*

*“I also sometimes feel that he starts a sentence and just loses track of what he was going to say… Sometimes it just tails off, and it’s like he can’t even remember that he started the sentence.” Caregiver2*

*“It’s irritating my, my … sometimes my brain moves faster than my mouth and sometimes my mouth moves faster than my brain. And – but I’m aware of the slowing down.” PwP13*

1. **Shift in Social Life**

**Shrinking World**

*“Many people give up on conversation with him, because they just can’t wait long enough for his answer.” Caregiver2*

*“Normally, we’re sort of out quite a lot. Doing things. But I’ve been feeling quite restricted” PwP4*

*“Because this can sometimes happen in the most embarrassing situations, like in a shop or wherever. Well, my mother doesn’t go to shops any more; she’s too old. But, you know, in those kind of situations. And then another time she said, I can’t stand any more, because her brain had told her she can’t stand, and she’d just sit on the floor.” Caregiver10*

*“And whole conversations on the telephone I’m not too keen on because it relies on memory to a certain degree. And if I’m trying to remember or make arrangements with somebody just to do something or to chat what we’ve been doing … it’s not easy on the telephone.” PwP4*

*“Since [PWP]’s been ill, we haven’t seen anybody, because she’s been ill, and I think they’re – they always ask how she is, but they can’t say ‘oh do you want to go away for a weekend’ because they feel awkward with whether [PWP] could cope or not.” Caregiver3*

*“Because of mobility problems it’s difficult to get out and about… I mean really, his world does gradually shrink, and I think the things that – he still reads, he still listens to the radio. So he is still quite connected to the world. But his physical world has shrunk. Unless I take him out you know.” Caregiver2*

*“Is it fair to say when you gave up driving, it was devastating?” Caregiver11; “Yes […] Want the freedom” PwP11*

*“I mean, he was playing golf at the very beginning of last year, and then that got difficult because he couldn’t face the right direction, couldn’t understand the instructions, and we were getting worried he was going to hurt somebody by hitting the ball in the wrong direction.” Caregiver12*

*“…but he finds it more difficult, now, I think, to travel. If we want to go long-haul on an aircraft or anything, he finds it… that to him now - where he used to love it, I think, you… I think he’s kind of scared of it, now.” Caregiver4*

*“…that was the last cruise we did. But he kept getting lost on the boat… and once he went missing and I don’t know where he was, and it was a nightmare... So, it was really difficult. But it’s then that I realised, he couldn’t cope with anything, any strange environment.” Caregiver12*

*“You know what people with Parkinson’s, when they become withdrawn. When they become alone. I think that’s very damaging to them. The human interaction is missing and that’s what needs to be there. I know some people get very, very depressed and then suffer with depression, during the Parkinson’s diagnosis and then on, but if you have somebody behind you to push you, or to take you to these activities, you will get involved.” HCP4 (PDNS)*

*“I think a lot of time they're probably quite lonely, feel quite isolated as well, and just need that human contact.” HCP15 (GP)*

*“Because obviously one of the biggest problems that people with memory problems have is motivation, and more often than not it’s that starting an activity. And obviously sometimes, getting lost halfway through and potentially sometimes finishing an activity. Which obviously all have an impact on that person’s ability, but also there are certainly risks associated with them as well.” HCP5 (OT, memory service)*

*“So, the memory, yes, it’s … I think it’s from not mixing, as well, with people, that you become … lose your memory more. If I’m right.” PWP9*

**Intensified Relationships**

*“I don’t like to do my bills or anything on the computer. So, my son, in one sense, has taken over, which I didn’t want him to.” PwP9*

*“Because at the end of the day I suppose, I don’t like to say this, but she’s [wife] my carer. If not now then certainly will be.” PwP5*

*“I think I had taken over the finances, sort of at least not managing them, just keeping an eye on them. I don’t do buying and selling. I just leave things as they are on the basis that if they go down, they’ll go up again. [laughs] Which distresses him a lot, because he thinks he can still do it. He still wants to do it, but I know I can’t; it would be inappropriate” Caregiver12*

*“I mean I do find the speech difficult for me as well. Because it makes any interaction with him very lengthy.” Caregiver2*

*“And, the most distressing, I think, are the ones that have a lack of insight and awareness to the extent they’ve changed but their family members really struggle.” HCP18 (Neuropsychologist)*

*“I think sometimes I’m overburdening him [husband] with things that he might not want to be doing.” PWP4*

*“Because he [another PwP in family] thought that I was mum, I was his daughter. So, he was just getting so confused. And you know, like mum said, even then, you can’t blame him, because it’s not him. It’s the illness... So, and it hurt.” Caregiver14*

*“one thing that particularly carers can find upsetting is apathy. So, if their partner used to be a busy kind of person and interested in lots of things and then they become apathetic, that can be quite trying for a partner.” HCP17 (geriatrician)*

*“…it can be really difficult when it’s all the time, every single conversation that you’re saying, and you can’t understand what a person is saying to you.” HCP20 (Speech & Language Therapist)*

*“And then, I think also in that transition where it’s a person who’s got Parkinson’s and they’re progressing, and they’re at home and they need more care and they’re starting to wander at nights or shouting and the wife is really struggling. So, that transition where you think things are getting harder at home, managing that where I’ve got quite a few patients like that, that is quite difficult.” HCP19 (neurologist)*

*“And there was a time when I was up two or three every night, or every hour or two hours with him, but they’ve given him knock-out jobs now so [laughs] I can get a night’s sleep now.” Caregiver12*

1. **Living Well**

**Intrinsic Motivation**

*“I can just get on and manage it myself. I’ve always been very self-reliant, so having Parkinson’s is just something that’s there and I have to embrace and get on with it.” PwP8*

*“But I’m also one very much in favour of managing Parkinson’s. I think, you know, yes we need to find out a lot of things about how it’s caused and the rest of it, but from the research side, some of the research, like what you’re doing, how to cope is important as anything. You know, people live 15-20 years with this. Their life would be much better if they’re helped in certain ways or if they help out themselves in certain ways.” PWP1*

*“…it takes a lot of concentration… It’s positive action, mind over matter.” PwP13*

*“But I don’t give up” PwP9*

*Offspring caregivers (see manuscript for example of spouse) “I mean, she’s my mum, you know? She looked after me, now it’s my turn to look after her. But it’s hard. [crying] It’s really hard. Because, you know, I know it’s not her, that it’s the disease … but I’m here with her 24/7, and it’s hard to see. And from – going back to my childhood, knowing what she was like then to now, it just kills me. It’s so, so sad.” Caregiver14*

**Self-Management Strategies**

*“The other thing is in the last week [PwP] has started seeing people….* *Well, I did know and have actually met people, because we belong to support groups, or have done, and I have met a person, a retired reverend, who had a whole crowd of people in with him all the time. And also, we’ve done a lot of reading […] And so when this happened about five days ago, I went on the Parkinson’s disease site […] A huge amount of help […] Then I spoke to the neurological nurse locally, who we know, then I spoke to our GP” Caregiver11*

*“I think they use a routine a lot. So, in those I think are managing better, they use a routine and just are well… […] I remember a gentleman, he goes out every day for a walk, he does it at 6 o’clock. He sticks to his routine, he does little exercises, and just watches his diet and attends appointments.” HCP3 (Dementia Nurse Specialist)*

*“I mean I manage it with, I’ve got a pill box, I’ve got a pill box that goes off every 4 hours or so. I’ve got my exercises that I try to do daily as I say. And that’s quite a routine” PwP1*

*“You need tools to do things with. You can’t just sit with – well, you sit with the shock of it, but you need tools.” Caregiver11*

*[And when you’re finding it more difficult, what do you do?] “Concentrate.” PwP13*

*“I’ve got – religiously – I have got my diary, which I keep next to me. Because I have done [forgotten appointments] a couple of times, which I hate myself, that I forget, so. And now, every morning, I pick up my diary as I get out of bed, […] and go through to make sure that I’ve got everything written down. And I re-check myself, again.” PwP9*

*“So, basically what we do is we give them [‘ABC charts’] to family members and carers. And we say to them, ‘look, would you mind filling in these charts?’ And it’s sometimes like I say, family members are more likely to say that they’re learned things about themselves, and things which have been positive and negative that they possibly won’t do again.” HCP5 (OT, memory service)*

*“So, I kind of remember the things I think are important, but switch off a bit with things that I think are less important.” PwP8*

**Supportive Relationships**

*“Because there's so much informal care that they provide, and if they didn't it would just be unmanageable, I think, for some of the patients and they’d end up in care facilities or care homes, nursing homes, things like that.” HCP13 (GP)*

*“I probably talk to my friend. […] And we talk – because he knows all my problems so he’s the only person that- And he’s got something to do with prostate and things like that. So he’s got a problem, so he can talk about his with me, and we talk about his prostate, I talk about my Parkinson’s.” PwP15*

*“Usually, we put Parkinson’s magazine on the coffee table as we get it out a lot. Most of our friends who call in, you know, there’s this magazine to look at while they’re here or when they go […]. You know, it helps them and it helps us. It gives them guidance on issues for looking after and helping us if needs be.” PwP4*

*“So, it was quite a good life, considering how bad he [person at peer support] had it. I mean, he got to the stage he was completely bent over and what have you, but he still carried on, and he was very encouraging, so. If you see people like that, you feel more … happier, yourself. Kind people.” PwP9*

*“But I suppose it’s [support group] also made me feel that I’m not the only one” Caregiver12*

1. **Preconceptions about Cognitive Impairment**

*“I’m aware that it’s common for people to get cognitive impairments or dementia with Parkinson’s. But I don’t think that’s always talked about, because there’s obviously lots of other things that are covered with a neurologist around Parkinson’s” HCP2 (Psychiatrist)*

*“it’s [dementia diagnosis] met with disbelief. “I’m not that person; why are you trying to put me in the bracket of that person?”” HCP3 (Nurse Practitioner, Memory Service)*

*“…[PwP] want to minimise any difficulties, even if at some level they know that there’s a problem.” HCP6 (Speech & Language Therapist)*

*“I’m quite honest about that. I don’t think anybody really understands this type of Parkinson’s…” Caregiver10*

*“I don’t think my comprehension has been damaged, well it probably has to some extent, but I don’t think it’s been greatly damaged. But that’s it, really… I don’t feel that I want to open up that can of worms at the moment.” PwP13*

*“No, I haven’t and I haven’t even discussed that [cognitive decline] with [wife], because that could lead onto other things and I really don’t want to think about that.” PwP5*

*“I couldn’t draw it, and so I got frustrated. She [Nurse] said, ‘Don’t. You haven’t got Alzheimer’s, yet.’ So, I said, ‘I hope I don’t get it.’ That’s what frightens me even more so. Because I’m forgetting names, and … I had a very good memory, to be honest with you.” PwP9*

*“…there is a big stigma still about dementia, people do not want the dementia diagnosis or label over them.” HCP22 (PDNS)*

*“And, it’s interesting because with some people with Parkinson’s disease do, kind of, put down because it’s targeting older age, so they kind of say, oh, it’s down to age the fact I can’t remember certain things, or I can’t, you know. Actually, it’s sometimes, yes, indeed, age has something to do with it, but there could be other factors.” HCP16 (PDNS)*
